# A Novel Animal Model for Regional Microbial Dysbiosis of the Pioneer Microbial Community

**DOI:** 10.3389/fmicb.2019.01706

**Published:** 2019-07-24

**Authors:** Nilusha Malmuthuge, Philip J. Griebel

**Affiliations:** ^1^Vaccine and Infectious Disease Organization-International Vaccine Centre, University of Saskatchewan, Saskatoon, SK, Canada; ^2^School of Public Health, University of Saskatchewan, Saskatoon, SK, Canada

**Keywords:** animal model, microbial dysbiosis, pioneer microbiota, gut micobiota dysbiosis, pioneer microbial communities, intestinal regionality, microbial restoration

## Abstract

Pioneer microbiota colonizing the newborn gastrointestinal tract has long-lasting effects on host health. Restoration of the gut microbial community, following dysbiosis during the neonatal period, may be one strategy to prevent undesirable health outcomes linked to an altered neonatal gut microbiome. Without appropriate animal models that recreate the prolonged human neonatal developmental period it is not possible to effectively analyze interventions designed to restore regional microbial populations. Our study used a lamb model in which intestinal segments were surgically isolated (blind-ended) in fetal lambs to create early microbial dysbiosis by delaying post-natal exposure to intestinal ingesta. Intestinal segments isolated *in utero* retained blood flow, innervation, and lymphatic drainage through the mesenteric attachment. Continuity of the fetal gastro-intestinal tract was re-established by side-to-side anastomosis of intestine proximal and distal to each isolated intestinal segment. Microbial restoration was then implemented in neonatal lambs by reconnecting a portion of the *in utero* isolated intestinal segments to adjacent intestinal tract 1 and 7 days after birth. Bacterial communities colonizing the adjacent intestine, *in utero* isolated intestinal segments, and reconnected intestinal segments were profiled using 16S amplicon sequencing on days 1, 7, and 56 of age. The *in utero* isolated intestinal segments were colonized 1 day after birth but the density of active bacteria was reduced and community composition altered when compared to adjacent intestine. *Proteobacteria* dominated the adjacent small intestine at early time points (day 1 and day 7) with a shift to primarily *Firmicutes* on day 56, consistent with establishment of an anaerobic bacterial community. In contrast, *Proteobacteria* persisted as the predominant community for 56 days in the *in utero* isolated intestinal segments. There was, however, almost full restoration of the microbial community composition in the *in utero* isolated intestinal segments following reconnection to the adjacent intestine. The density of beneficial bacteria, especially *Bifidobacterium*, remained significantly lower in the reconnected intestinal segments at 56 days when compared to adjacent intestine. Post-natal persistence of a stable pioneer community (*Proteobacteria*) in the *in utero* isolated intestinal segments provides a model system to study the temporal effects of regional microbial dysbiosis throughout a prolonged neonatal period.

## Introduction

The pioneer microbiota colonizing the newborn gastrointestinal tract (GIT) has long-lasting effects on host health and early microbial dysbiosis (altered abundance, composition, and diversity) has been linked to allergy, asthma, obesity, and inflammatory bowel diseases (Dominguez-Bello et al., 2016; Milani et al., 2017). Increased urbanization (formula/bottle feeding, lack of maternal, and environmental exposure) and early medical interventions (cesarean deliveries, antibiotics) are factors that affect the human infant microbiota (Rampelli et al., 2015; Dominguez-Bello et al., 2016; Ayeni et al., 2018). Similarly, early feeding practices (Malmuthuge et al., 2015; Maynou et al., 2016, 2017; Song et al., 2019), antimicrobials (Oultram et al., 2015; Pereira et al., 2016), and oral supplements (Foditsch et al., 2015) have been reported to influence the gut microbiota of neonatal ruminants. Restoration of the gut microbial community, following dysbiosis during the neonatal period, may be one strategy to minimize the risk of undesirable health outcomes associated with an altered neonatal gut microbiota. A recent study partially restored cesarean-born infant gut microbiota by exposing newborns to microbiota from the maternal birth canal (Dominguez-Bello et al., 2016), indicating early microbial restoration is possible. However, the impact of these early interventions on regional microbial populations, development of mucosal immunity in the GIT, and systemic health have yet to be fully analyzed. These analyses have been limited by a lack of appropriate animal models that recreate the prolonged developmental period of human infants and the lack of methodology to identify significant relationships between the microbial community and functional changes in the neonatal mucosal immune system.

The composition of gut microbes varies significantly throughout the GIT and between the intestinal lumen and mucosal surface (Malmuthuge et al., 2012, 2014; Romano-Keeler et al., 2014). These studies support the conclusion that fecal microbiota is not representative of microbiota throughout the GIT. Thus, it is essential to study microbiota within specific intestinal regions to fully understand the impact of early microbial interventions. The analysis of regional enteric microbiota presents substantial challenges, since it requires access to the small intestine, which may be limited by ethical issues or a lack of suitable models and tools. Newborn ruminants have successfully been used to study host responses to regional gut microbiota through construction of surgically isolated intestinal segments (Charavaryamath et al., 2013; Maattanen et al., 2013).

Rodent models are widely used to understand molecular mechanisms mediating host-microbial interactions in the gut. Discoveries based on rodent models revealed that the host is aware of the intestinal microbiota through continuous sensing/sampling of the intestinal content and this process is of great importance to regulate intestinal homeostasis. Recently, a site-specific microbial regulation of the mucosal immune system has also been reported using a mouse model (Sommer et al., 2015). This study further revealed that microbiota colonizing the ileum mainly influenced immune functions, whereas microbiota colonizing the colon influenced the metabolic functions of host. Mice, however, are born with an underdeveloped mucosal immune system in the small intestine when compared to humans and have a very brief neonatal period (Hein and Griebel, 2003). In contrast, newborn ruminants share many similarities with human infants in both pre- and post-natal development of the enteric mucosal immune system (Griebel and Hein, 1996; Hein and Griebel, 2003). For example, the small intestine of fetal humans and ruminants have well-developed T cell and B cell subsets, whereas few lymphocytes appear in the mouse intestines before birth. Thus, mice do not provide an effective model to identify mechanisms mediating the mucosal immune system’s first response to the colonizing microbiome and whether sustained dysbiosis throughout the neonatal period can have long-term effects on mucosal immune function. It is essential to develop an animal model with greater immunophysiological similarities to the human infant during the neonatal period as well as a model suitable for studying possible interventions to correct the dysbiosis.

The present study used newborn lambs in which intestinal segments were surgically prepared *in utero* when the fetus is sterile during the third trimester of pregnancy (Malmuthuge and Griebel, 2018). The intestinal segment retains its mesenteric attachment to maintain blood flow, innervation, and lymphatic drainage, while continuity of the GIT is re-established by a side-to-side anastomosis of the intestine proximal and distal to the isolated intestinal segment. The intestinal segment was then subdivided into multiple compartments in the newborn lamb and used to reconnect to the adjacent intact intestine. Stable regional microbial dysbiosis, characterized by an aerobic pioneer community, persisted throughout the neonatal period in the compartment that remained isolated from intestinal ingesta. Microbial restoration was implemented by reconnecting individual compartments of the isolated intestinal segment to the adjacent intact intestine at 1 and 7 days after birth. Microbial communities in the isolated and reconnected compartments were then compared over an 8 weeks period with the adjacent intact intestine. This model facilitated an analysis of age-matched changes in the microbiome within a genetically identical environment. Thus, a model system for persistent regional microbial dysbiosis in the neonatal small intestine was developed and used to study the microbial community following restoration with autochthonous microbiota from the same region of the intestine.

## Materials and Methods

### Creating Intestinal Segments *in utero*

All experimental protocols were reviewed and approved by the University of Saskatchewan Animal Care Committee (AUP20160105), which follows the guidelines of the Canadian Council on Animal Care. Estrous synchronization and timed mating ensured gestation time was known when surgeries were performed. Sheep were transported to the research facility and acclimated for 1 week prior to surgery. Fetal surgeries were performed (125–135 days of gestation) to create isolated intestinal segments 15–20 cm cranial to the ileo-cecal fold (Mutwiri et al., 1999; Tsang, 2007). Sterility was maintained throughout surgery to prevent environmental contamination of the fetus or intestine (Malmuthuge and Griebel, 2018). Briefly, the ventral abdomen of the ewe was clipped, cleaned with surgical soap, and the incision site covered with a steridrape prior to opening the abdomen. The fetus was accessed through a uterine incision and fetal intestine was exposed through a fetal abdominal incision. Fetal intestine was occluded with two intestinal clamps placed at the proximal and distal end of the segment prior to making an incision between each set of clamps to ensure the intestinal lumen was not exposed to the external environment during surgery. A blind-ended intestinal segment (∼15 cm) was prepared cranial to the ileo-cecal junction (distal small intestine). This intestinal segment retained its mesenteric attachment to maintain blood flow, innervation, and lymphatic drainage but had no connection to the lumen of adjacent intestinal lumen. Continuity of the GIT was re-established by side-to-side anastomosis of the intestine proximal and distal to the isolated intestinal segment. This ensured the fetus was born with a functional GIT but the surgically created isolated intestinal segment was not exposed to maternal and environmental microbiota at birth (Mutwiri et al., 1999).

### Reconnecting Subsections of the Isolated Segment to the Adjacent Intact Intestines in Neonatal Lambs

**FIGURE 1:**
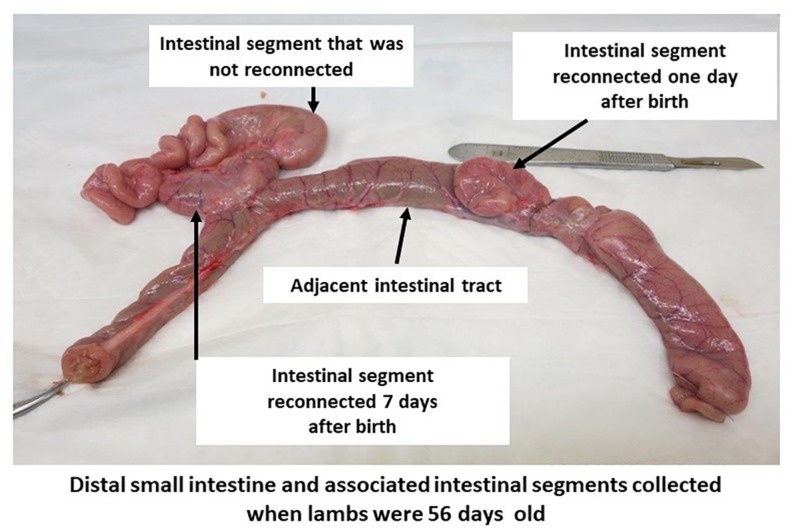
Intestinal segment subdivided into subsections in 56-day-old lambs. A segment of small intestine, with intact mesenteric attachment, was surgically isolated *in utero* during the third trimester of gestation. Subsections of this isolated intestinal segment was re-connected by side-to-side anastomosis to the adjacent small intestine on one and seven days after birth. A portion of the intestinal segment isolated *in utero* remained separated from the intestinal tract throughout the 56 days post-partum period.

Two intestinal surgeries (1 and 7 days after birth) were performed following the birth of each lamb to reconnect subsections of the *in utero* isolated intestinal segment to adjacent intact intestine. Lambs were anesthetized and prepared for each surgery as described previously for the ewes (Malmuthuge and Griebel, 2018). The first surgery was performed within 24 h after birth to perform a side-to-side anastomosis of a 5–10 cm subsection of the *in utero* surgically isolated intestinal segment to the adjacent intestine (Figure 1). Prior to performing each anastomosis, intestinal tissue and contents were collected from the adjacent intact intestine (N1) and *in utero* isolated intestinal segment (S1). Briefly, a 5–10 cm subsection of the intestinal segment isolated *in utero* was first separated with two intestinal clamps prior to making an incision between each set of clamps to ensure the isolated intestinal segment was not exposed to the external environment during surgery. Then, the separated subsection of the intestinal segment was connected by a side-to-side anastomosis to the adjacent intact intestine to facilitate intestinal content flow into the *in utero* isolated intestinal subsection. Recovered lambs from the first intestinal surgery were then subjected to a second intestinal surgery 7 days after birth. A second 5–10 cm subsection of the original *in utero* isolated intestinal segment was connected by side-to-side anastomosis with adjacent intact intestine when lambs were 7 days old (Figure 1). Intestinal tissue and contents (adjacent intact intestine – N7, isolated intestinal segment – S7, intestinal segment reconnected on day 1 – S1R7) were again collected on day 7 prior to performing the anastomosis. The two intestinal surgeries performed after birth left a 5–10 cm length of the original *in utero* intestinal segment separated from the adjacent intact intestine throughout the 8 weeks post-natal period. All subsections of the intestinal segment retained a mesenteric attachment to maintain blood flow, innervation, and lymphatic drainage (Figure 1). When lambs were 56 days old, intestinal tissue and contents were collected from adjacent intact intestine (N56), the *in utero* intestinal segment that remained isolated throughout the experiment (S56), subsections of the *in utero* intestinal segment that were reconnected on day 1 (S1R56) and subsections of the *in utero* intestinal segment that were reconnected on day 7 (S7R56). All intestinal samples were snap frozen using liquid nitrogen and stored at −80°C until processed.

### Extraction of Nucleic Acids

Total genomic DNA was extracted using the repeated bead-beating plus column method (Yu and Morrison, 2004). Briefly, 0.1–0.5 g of ground intestinal sample (tissue and content of adjacent, isolated, and reconnected intestinal segments) was subjected to bead-beating twice in 2 mL screwcap tubes containing 0.5 mm Zirconium beads and using a Mini-beadbeater-16 (BioSpec Products Inc., Bartlesville, OK, United States). Impurities and SDS used during bead-beating steps were removed with ammonium acetate and nucleic acids precipitated with isopropanol. Finally, recovered nucleic acids were purified using QIAamp Fast DNA Stool Mini Kit (QIAGEN, Germantown, MD, United States) following the manufacturer’s instructions. Extracted DNA was quantified using ND 1000 spectrophotometer (NanoDrop Technologies, Wilmington, DE, United States) as well as Qubit 3.0 fluorometer and DNA HS array kit (Thermo Fisher Scientific, Waltham, MA, United States).

Total RNA was extracted using mirVana miRNA isolation kit, with phenol (Invitrogen, Carlsbad, CA, United States) following the manufacturer’s instructions. Briefly, 0.05–0.07 g of ground intestinal sample was homogenized using PreCelly’s CK14 Lysin kit (Bertin Technologies, Montigny, France) with added lysis buffer. Following incubation with miRNA homogenate additive and acid-phenol:chloroform, RNA was precipitated using cold ethanol. The quantity of RNA was measured using Qubit 3.0 fluorometer and RNA HS array kit (Thermo Fisher Scientific, Waltham, MA, United States) and RNA integrity number (RIN) was measured using a Bioanalyzer 2100 (Agilent Technologies, Santa Clara, CA, United States).

### Profiling of Intestinal Bacterial Community Using 16S Amplicon Sequencing

Diluted genomic DNA (25 ng/μL) was subjected to a nested PCR using the 27F (3′-AGAGTTTGATCMTGGCTCAG-5′) and 1492R (3′-TACGGYTACCTTGTTACGACTT-5′) primer pair (Weisburg et al., 1991), followed by 27F (ACACTGACGACATGGTTCTACA-3′-AGAGTTTGATCMTG GCTCAG-5′) and 519R (TACGGTAGCAGAGACTTGGTCTCC-3′-GCGGCKGCTGGCAC-5′) universal bacterial primers containing tags to profile total bacterial community using 16S amplicon sequencing. Diluted genomic DNA was also subjected to another nested PCR using the 27F (3′-AGAGTTTGATCMTGGCTCAG-5′) and 1492R (3′-TACGGY TACCTTGTTACGACTT-5′) primer pair (Weisburg et al., 1991), followed by the genus specific BIF-164F (ACACTGACGACATGGTTCTACA-3′-GGGTGGTAATGCCG GATG-5′) and BIF-662R (TACGGTAGCAGAGACTTGGTC TCC-3′-ACCGTTACACCGGGAA-5′) primer pair (Satokari et al., 2001) containing tags to profile the bifidobacterial community. Both sequencing runs included no template controls (only reagents used for PCR) that went through the same PCR and barcoding process. All PCR products were sequenced at Genome Quebec (McGill University, Quebec, Canada) using MiSeq sequencing platform (Illumina, San Diego, CA, United States). Raw sequences were deposited in the NCBI Sequence Read Archive (SRA) under the accession number PRJNA507407^1^.

### Analysis of 16S Amplicon Sequencing Data

Sequence data were analyzed using the Quantitative Insights Into Microbial Ecology (QIIME) software version 2 (Bolyen et al., 2018). First, quality histograms were generated from forward and reverse sequences to determine positions at which raw sequences needed to be truncated based on Phred quality score. Denoising, joining paired ends, and quality filtering to remove chimeric sequences were performed using DADA2 within QIIME with qiime dada2 denoise-paired command. During this step, forward sequences were trimmed at 275 bp and reverse sequences were trimmed at 280 bp to retain sequences with a Phred quality score > 25. Then, any remaining primer sequences were removed from the joined sequences using cutadapt version 1.16 (Martin, 2011). The remaining high quality sequences were used to perform operational taxonomic units (OTUs) clustering at 97% similarity using SILVA 132 QIIME release and qiime vsearch cluster-features-closed-reference command. OTUs identified from the no template controls were then filtered from samples using qiime feature-table filter-features command. Remaining OTUs were used for taxonomic assignment using the SILVA 132 97% classifier. α- and β-diversity analyses were also performed within QIIME to explore the diversity of intestinal bacterial community within each intestinal segments and among intestinal segments, respectively. Community comparisons were performed using analysis of similarity (ANOSIM) and weighted UniFrac distance matrices within QIIME platform. Use of SILVA database within QIIME platform only classified sequences up to genus level. Therefore, representative sequences of the generated bifidobacteria and lactobacillus OTUs were then used to assign taxonomy at species level using BLAST algorithm in NCBI.

### OTU-Based Clustering of Microbial Profiles

A correlation-based hierarchical clustering approach (Malmuthuge et al., 2019) was used to explore similarities or dissimilarities of the total bacterial communities profiled from three intestinal segments. First, a Spearman rank correlation analysis was performed using the cor() function in R to obtain correlation (ρ) between each pair of small intestinal microbial profiles based on the relative abundance of OTUs clustered at 97% similarity. Then, a distance matrix constructed using ρ^2^ values was used to perform hierarchical clustering using the hclust() function within R package (V 3.4.1) to obtain clustering tendencies of intestinal microbial profiles. The observed microbial clusters were then validated by assessing the clustering tendency using the get_clust_tendency() function in the R packages cluster and factoextra to calculate Hopkins statistics. Approximately unbiased (AU) probability values (*p*-values) of the validated clusters were then calculated using a multiscale bootstrap resampling (1000 bootstrap replications) in the R package pvclust (Suzuki and Shimodaira, 2006) to identify distinct clusters.

The relative abundance of bacterial phyla between identified clusters was then compared using a non-parametric *t*-test (Mann–Whitney–Wilcoxon) in R (3.3.1v), and a multiple test correction was performed according to the method described in Benjamini and Hochberg (1995) to identify the differences associated with microbial clusters.

### Estimation of Active Bacterial Densities

Total RNA was used to synthesize cDNA using iScript reverse transcription supermix for RT-qPCR (Bio-Rad Laboratories Inc., Hercules, CA, United States) and cDNA was subjected to quantitative real-time PCR (qPCR). The HDA1 and HDA2 universal bacterial primer pair, *Bifidobacterium* and *Lactobacillus* genus-specific primers (Table 1), and SYBR green chemistry (Fast SYBR^®^ Green Master Mix, Applied Biosystems, Foster City, CA, United States) were used with a StepOnePlus real-time PCR system (Applied Biosystems, Foster City, CA, United States). Standard curves were constructed using serial dilutions of PCR products generated using each primer pair. Copy number of each standard curve was calculated based on the following equation; (NL × A × 10^–9^)/(660 × n), where NL – Avogadro constant (6.02 × 10^23^), A – molecular weight of DNA molecules (ng), and n – length of amplicon (bp). The copy number of 16S rRNA genes per gram of fresh sample was calculated using the following equation: (QM × C × DV)/(S × W) [QM – quantitative mean of 16S rRNA copies, C – RNA concentration of each sample (ng/μL), DV – dilution volume of extracted DNA (μL), S – RNA amount subjected to analysis (ng), and W – sample weight subjected to RNA extraction (g)] (Li et al., 2009).

**TABLE 1:** Bacterial primers used to estimate the density of active bacteria.

	**Primer sequences 3′-5′**	**References**
Total bacteria	HDA1 – ACTCCTACGGGAGGCAGCAGT HDA2 – GTATTACCGCGGCTGCTGCTGGCAC	Walter et al., 2000
*Bifidobacterium*	Bif1 – CGTCAAGCTGCTAGGACGC Bif2 – TACACCGGAATAGCTCCTGG	Liang et al., 2014
*Lactobacillus*	Lac1 – AGCAGTAGGGAATCTTCCA Lac2 – ATTTCACCGCTACACATG	Liang et al., 2014

Bacterial density data were first checked for normality using QQ-norm plots and Shapiro–Wilk normality test in R (3.3.1v) and log transformed data were then analyzed using a two-way unbalanced ANOVA in R (3.3.1v). The following statistical model was used test the effects of intestinal region and sampling time point on intestinal bacterial density; Y_*ijk*_ = μ + S_i_ + T_j_ + (ST)_ij_ + e_ijk_, where Y – bacterial density (total, *Bifidobacterium, Lactobacillus*), μ – mean, S – intestinal segment, T – sampling time, ST – intestinal segment by sampling time interaction, and e – residual error. Statistical differences were declared at *P* < 0.05 using Tukey HSD test and lsmeans() function in R.

## Results

### Animal Model for Regional Gut Dysbiosis

A model of regional enteric microbiota dysbiosis was developed by surgically creating isolated intestinal segments in the distal small intestine of fetal lambs during the third trimester of pregnancy. Six lambs with isolated intestinal segments were born alive and five of the six surgical lambs were from ewes carrying twins. The lambs underwent two intestinal surgeries to anastomose subsections of the intestinal segment isolated *in utero* on one and 7 days after birth. One lamb did not recover from anesthesia following surgery on day 1 and one lamb recovered but died 2 days after the first surgery. The remaining four lambs survived the second abdominal surgery and remained healthy throughout the 8 weeks post-natal period.

### Colonization of Adjacent Intact Intestine During the First Two Months of Life

**FIGURE 2:**
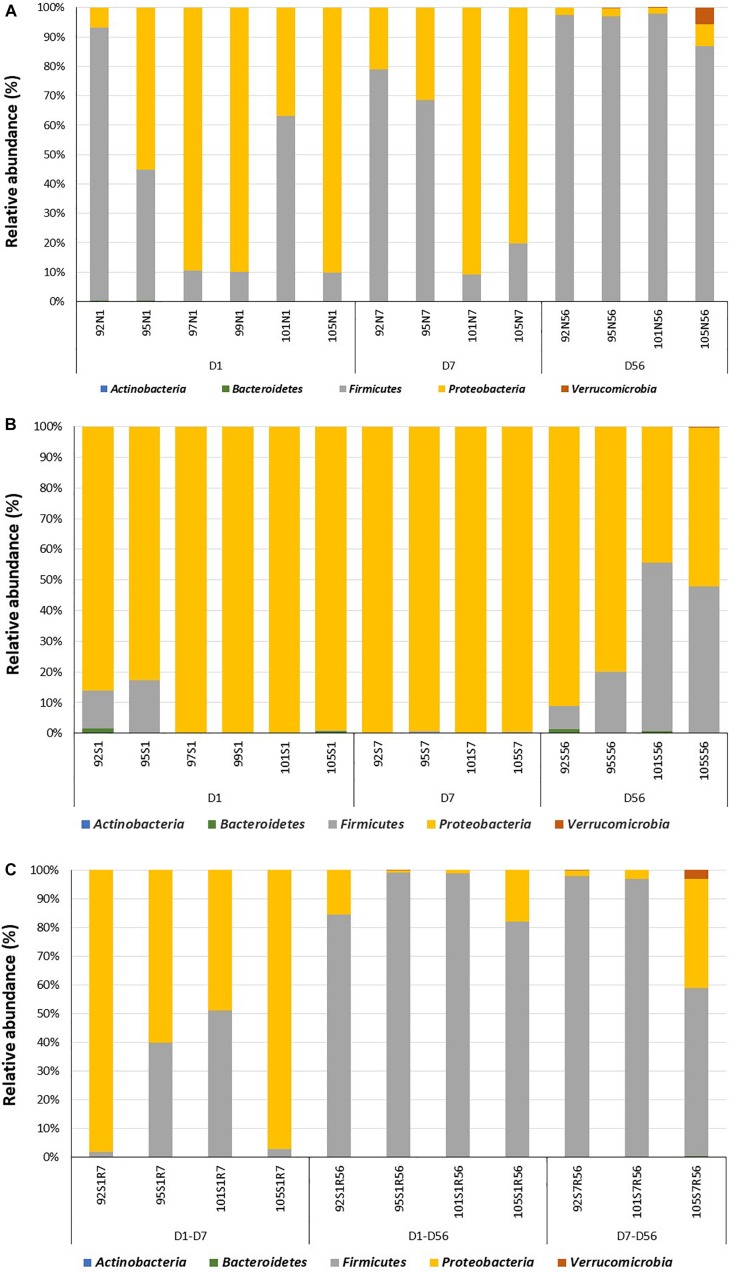
Colonization of the adjacent intact intestine and subsections of the surgically isolated intestinal segment during the first 2 months of life. **(A)** Microbial profiling of intestinal samples collected from adjacent intact intestine (N) on day 1 (N1), 7 (N7) and 56 (N56) post-partum. **(B)** Microbial profiling of the surgically isolated intestinal segment (S) that remained isolated on days 1 (S1), 7 (S7), and 56 (S56) post-partum. **(C)** Microbial profiling of the isolated intestinal segment subsection that were reconnected to the adjacent small intestine on day 1 post-partum and then sampled on day 7 (S1R7) and 56 (S1R56) post-partum. A second subsection of the intestinal segment was reconnected to the adjacent small intestine on day 7 post-partum and then sampled on day 56 (S7R56) post-partum. Each bar represents a sample collected from an individual animal (92, 95, 97, 99, 101, 105) at the time points indicated.

Amplicon sequencing was used to profile the intestinal bacterial community colonizing the lamb intestine after birth. In total, 390 OTUs were identified from all samples collected from normal intestine throughout the experimental period. The number of OTUs identified in day 1 samples (47 ± 11) was significantly lower (*P* = 0.02) when compared to samples collected on day 7 (82 ± 2) and day 56 (85 ± 6) of age. Bacterial populations in the distal small intestine of neonatal lambs contained 3 bacterial phyla, 15 bacterial families, and 17 bacterial genera (Figure 2A and Supplementary Figure 1A). *Proteobacteria* (63.4 ± 16.4%) dominated the bacterial community on day 1, followed by *Firmicutes* (36.5 ± 15.1%) and *Bacteroidetes* (0.10 ± 0.07%). At the genus level, the microbial community was dominated by *Massilia* (32.5 ± 10.3%), followed by *Lactobacillus* (28.3 ± 15.1%), *Escherichia*–*Shigella* (17.3 ± 11.2%), *Delftia* (9.5 ± 4.9%), and *Acinetobacter* (3.6 ± 1.0%) (Supplementary Figure 1A).

More complex bacterial communities were observed in the intact small intestine of lambs on day 7 and day 56 (Figure 2A and Supplementary Figure 1A). Bacterial diversity indices (Chao1, Observed species and PD whole tree) were numerically higher on day 7 and day 56 compared to day 1 (Supplementary Figure 1B). Anaerobic bacterial phyla, including *Actinobacteria* (day 7 – 0.04 ± 0.02%; day 56 – 0.15 ± 0.04%) and *Verrucomicrobia* (day 7 – 0; day 56 – 1.7 ± 1.3%) were detected in the intestine after day 7 of age. Moreover, the early *Proteobacteria*-dominant bacterial community (day 7 – 56.1 ± 17.6%; day 56 – 2.5 ± 1.1%) was displaced by *Firmicutes* on day 56 (day 7 – 43.9 ± 18.5%; day 56 – 95.6 ± 2.3%). In total, 17 and 25 bacterial families were identified on day 7 and day 56, respectively. Similarly, the number of identified bacterial genera increased from 16 on day 7–29 on day 56. *Escherichia*–*Shigella* (53.2 ± 16.4%) and *Lactobacillus* (42.3 ± 18.5%) dominated the small intestinal bacterial community on day 7, while *Lactobacillus* (64.5 ± 11.7%), *Streptococcus* (6.6 ± 3.4%) and *Escherichia*–*Shigella* (3.2 ± 1.1%) dominated the small intestinal bacterial community on day 56.

RNA-based quantification of active bacteria revealed a dense bacterial community in the distal small intestine of lambs, beginning at 1 day of age. Total bacterial density did not change significantly with increasing age (Table 2).

**TABLE 2:** Density of active bacteria colonizing the adjacent intact intestine and the subsections of the intestinal segment remaining either isolated or reconnected to the adjacent small intestine of neonatal lambs during the first 2 months of life.

Bacterial density^*^	**Day 1**		**Day 7**			**Day 56**			**SEM**	***P*-value**		
	**Adjacent**	**Isolated**	**Adjacent**	**Isolated**	**Reconnected**	**Adjacent**	**Isolated**	**Reconnected**		***P*_*S*_**	***P*_*T*_**	*P*_S*T_
Total bacteria	10.3^a^	9.7^b^	10.5^a^	9.7^b^	9.5^b^	10.4^a^	9.8^b^	10.3^a^	0.17	0.01	0.78	< 0.01
*Bifidobacterium*	8.9^a^	6.9^b^	8.9^a^	6.5^b^	6.4^b^	8.6^a^	6.5^b^	7.3^c^	0.19	< 0.01	0.10	0.02
*Lactobacillus*	7.3^a^	6.3^b^	7.6^a^	5.6^b^	6.1^b^	7.2^a^	6.3^b^	6.9^ab^	0.20	< 0.01	0.04	0.02

^*^Bacterial density is presented as log_10_ 16S rRNA copy/g of intestinal samples. ^a,b,c^The presence of a different superscript indicates that bacterial densities are differed at P < 0.05. P_S_, effect of intestinal segment; P_T_, effect of timepoint; P_S*Teffect_, of intestinal segment by timepoint interaction.

### Surgically Isolated Intestinal Segments Provide a Stable Environment to Study Microbial Dysbiosis

Surgically isolated intestinal segments were colonized with active bacteria within 24 h of birth (Figure 2B and Table 2). Estimation of active bacterial density revealed that isolated segments had lower (*P* = 0.01) bacterial density than normal (intact) intestine during the experimental period and bacterial density remained constant in isolated segments throughout the 56 days period (Table 2).

When the bacterial community was profiled, 370 OTUs were identified in all samples collected from the isolated intestinal segments. The number of OTUs identified on day 56 (80 ± 12) was significantly (*P* < 0.01) higher than the number identified on both day 7 (60 ± 3) and day 1 (25 ± 4). Similar to the normal (intact) intestine, bacterial diversity indices (Chao1, Observed species and PD whole tree) were numerically higher on days 7 and 56 when compared to day 1 (Supplementary Figure 1B). Taxonomic assignment revealed 3, 4, and 5 bacterial phyla on days 1, 7, and 56, respectively (Figure 2B). *Proteobacteria* dominated the isolated intestinal segments bacterial community throughout the entire experimental period (Figure 2B: day 1 – 92.5 ± 3.6%; day 7 – 98.5 ± 0.3%; day 56 – 65.3 ± 13.5%). A significantly (*P* < 0.05) higher abundance of *Firmicutes* (day 1 – 7.0 ± 5.3%; day 7 – 1.4 ± 0.1%; day 56 – 34.0 ± 12.6%) was, however, observed on day 56 compared to early time points. In total, 32 bacterial genera were detected in samples collected from all isolated intestinal segments (Supplementary Figure 1A). Among the 11 bacterial genera detected on day 1, *Massilia* (54.2 ± 3.2%), *Escherichia*–*Shigella* (18.5 ± 6.4%), and *Delftia* (15.3 ± 5.0%) dominated the isolated intestinal segments. *Escherichia*–*Shigella* (94.7 ± 1.6%) dominated the isolated intestinal bacteria at the genus level on day 7 (total 10 genera). On day 56, *Escherichia*–*Shigella* (94.4 ± 1.2%) and *Lactobacillus* (6.6 ± 2.9%) were the most abundant among the 29 identified bacterial genera.

**FIGURE 3:**
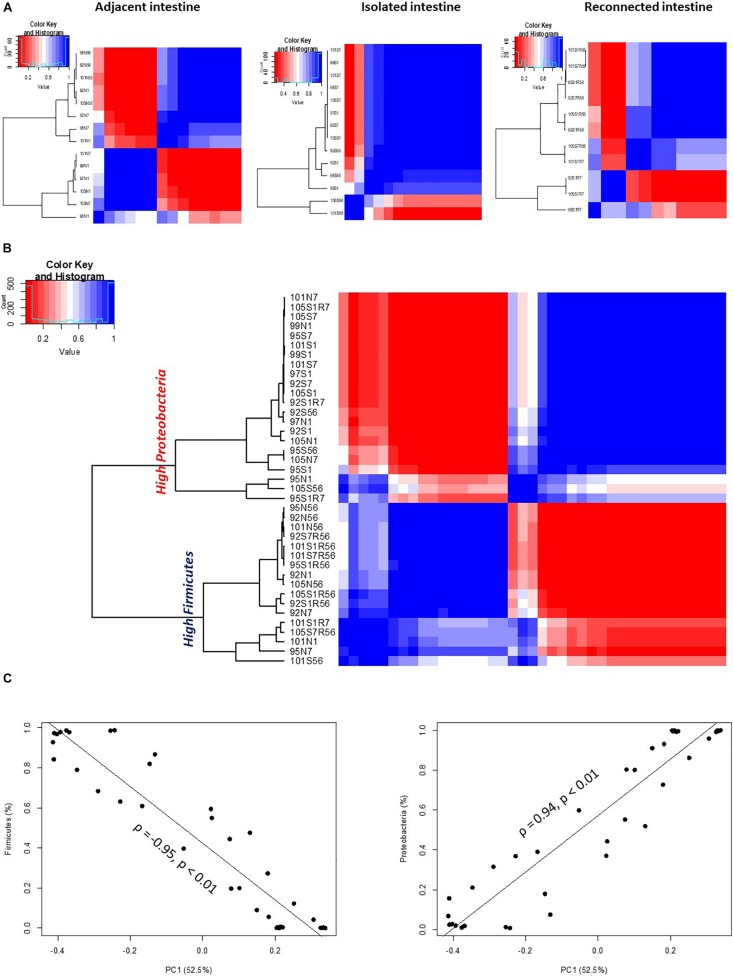
OTU-based clustering of microbial profiles generated from lamb intestinal samples. First, pairwise comparisons were made between individual microbial profiles and then hierarchical clustering using ρ^2^ was performed to explore similarities among the intestinal microbial profiles. Blue color represents highly correlated (ρ^2^ > 0.9, *P* < 0.05) microbial profiles. **(A)** Hierarchical clustering of microbial profiles within each subsection of the intestinal segments and adjacent intact intestine. The majority of microbial profiles generated from the intestinal (except 2) subsections that remained isolated from adjacent intestine belong to one main cluster (ρ^2^ > 0.9, *P* < 0.05), while profiles generated from adjacent intact small intestine and subsections of the intestinal segments reconnected to adjacent small intestine displayed varying clustering patterns. **(B)** Hierarchical clustering of microbial profiles generated from all intestinal samples resulted in two distinct clusters. Cluster 1 included all samples with a high relative abundance of *Proteobacteria* and Cluster 2 included all samples with a high relative abundance of *Firmicutes*. **(C)** Correlation between principle coordinate 1 (PC1) and the relative abundance of *Firmicutes* and *Proteobacteria*.

An analysis of the similarities or dissimilarities of the bacterial communities identified in isolated intestinal segments revealed these bacterial profiles were highly similar to each other, regardless of sampling time, except two outlier communities generated from day 56 samples (Figure 3A). High similarity among microbial profiles, regardless of the time point, also revealed the maintenance of a stable bacterial community in the isolated intestinal segments over the 8 weeks experimental period. When all bacterial profiles were evaluated, profiles generated from the isolated intestinal segments clustered with those generated from the earlier time points (day 1 and day 7) of adjacent intestine (Figure 3B). Correlation based hierarchical clustering of bacterial profiles generated within each animal also revealed a similar clustering pattern, where bacterial profiles of the isolated intestinal segments clustered with day 1 and day 7 profiles of the adjacent intestine (Supplementary Figure 2G). The cluster containing microbial profiles from the isolated intestinal segments and early time points (day 1 and day 7) of adjacent intestine had a higher abundance of *Proteobacteria* and lower abundance of *Firmicutes* when compared to the other cluster, which was comprised of profiles generated from day 56 adjacent intestine and reconnected intestinal segments (Figure 3B). Correlation between the first principle coordinate (PC1) and the relative abundance of bacterial phyla revealed that PC1 was positively correlated with the abundance of *Proteobacteria* and negatively correlated with the abundance of *Firmicutes* (Figure 3C).

Similar clustering patterns were also observed through beta-diversity analysis comparing microbial communities using UniFrac distance matrix and analysis of similarity (Supplementary Figure 2A and Table 3). Bacterial profiles generated from isolated intestinal segments shared fewer OTUs with those generated from the adjacent intestine on days 56 (ANOSIMR = 0.9768, *P* = 0.02) compared to day 1 (ANOSIMR = 0.3646, *P* = 0.01) and days 7 (ANOSIMR = 0.2810, *P* = 0.09) (Supplementary Figures 2B,C and Table 3). Similarly, bacterial profiles of the isolated intestinal segments on day 56 shared none or few OTUs with the reconnected intestinal segments analyzed on day 56 (Supplementary Figure 2D and Table 3).

**TABLE 3:** Comparison of bacterial profiles using analysis of similarity (ANOSIM).

Comparison^*^	**ANOSIM R**	***P*-value**
Time points (all regions)	0.4513	<0.01
Intestinal regions (all time points)	0.1969	<0.01
Intestinal regions (D7)	0.1366	0.12
Intestinal regions (D56)	0.4757	<0.01
Isolated vs. Adjacent (D1)	0.3646	0.01
Isolated vs. Adjacent (D7)	0.2810	0.09
Isolated vs. Reconnected (D7)	0.2708	0.07
Adjacent vs. Reconnected (D7)	−0.1146	0.56
Isolated vs. Adjacent (D56)	0.9768	0.02
Isolated vs. Reconnected (D56)	0.7735	<0.01
Isolated vs. Reconnected D1 (D56)	1.0	0.02
Isolated vs. Reconnected D7 (D56)	0.6111	0.05
Adjacent vs. Reconnected (D56)	−0.0555	0.56
Adjacent vs. Reconnected D1 (D56)	0.0208	0.40
Adjacent vs. Reconnected D7 (D56)	−0.0555	0.51
Reconnected D1 vs. Reconnected D7 (D56)	−0.0925	0.67

^*^Comparisons performed using weighted UniFrac distance matrix.

### Reconnecting Subsections of the Segment Isolated *in utero* to the Adjacent Intestine Re-establishes the Bacteria Community

Reconnecting isolated intestinal segments to adjacent small intestine facilitated bacterial colonization of the isolated segments by 56 days, regardless of the delay in connection after birth. The active bacterial density observed in intestinal segments reconnected on day 1 and sampled on day 7 remained similar to the isolated intestinal segments (Table 2). However, intestinal segments reconnected on both days 1 and 7 post-partum and sampled on day 56 had bacterial numbers similar to the functional adjacent intestine (Table 2).

Reconnecting isolated segments also influenced bacterial diversity (380 OTUs identified from all reconnected intestinal segment samples). In total, 189 OTUs (85 ± 7) were identified when day 1 reconnected intestinal segments were sampled on day 7 (S1R7). The number of OTUs identified from the S1R7 intestinal segments was significantly (*P* < 0.01) higher when compared to samples collected from the isolated intestinal segments on day 1 and tended (*P* = 0.06) to be higher than day 7 isolated intestinal segments. There was, however, no significant difference (*P* = 0.9) between the S1R7 intestinal segments and day 7 adjacent intestine. On day 56, the number of OTUs in samples collected from the day 1 reconnected segments (S1R56) was 62 ± 3 (total 169), while it was 83 ± 11 (total 197) in the day 7 reconnected segments (S7R56). The number of OTUs identified in all the reconnected segments on day 56 was not statistically different from those of day 56 adjacent intestine and day 56 isolated intestinal segments.

When bacterial composition was evaluated at the phylum level (Figure 2C), the S1R7 reconnected intestinal segments still had a higher abundance of *Proteobacteria* (76.9 ± 14.3%), followed by *Firmicutes* (22.8 ± 13.9%). On day 56, the S1R56 reconnected segment had a higher abundance of *Firmicutes* (91.1 ± 3.6%), followed by *Proteobacteria* (8.6 ± 4.2%). Similarly, the S7R56 reconnected segments were dominated by *Firmicutes* (87.3 ± 12.2%) and *Proteobacteria* (11.3 ± 9.8%) on day 56. Other bacterial phyla, *Actinobacteria*, *Bacteroidetes*, and *Verrucomicrobia* were also observed in both the S1R56 and S7R56 reconnected segments on day 56 (Figure 2C). *Escherichia*–*Shigella* (70.5 ± 13.5%) and *Lactobacillus* (41.5 ± 15.5%) dominated the S1R7 and S1R56 reconnected segments, respectively (Supplementary Figure 1A). While, *Lactobacillus* (43.5 ± 20.6%) dominated the S7R56 reconnected segments on day 56 (Supplementary Figure 1A). Once again, individual animal variation was greater in the reconnected intestinal segments when compared to the intestinal segments that remained isolated (Figure 3A).

When bacterial profiles generated from adjacent and reconnected intestinal segments were compared, it was evident they shared the majority of OTUs and were highly similar to each other (Figure 3B, Supplementary Figure 2E, and Table 3). Reconnection of the isolated segment facilitated establishment of bacterial communities similar to those in the adjacent functional intestine, regardless the postnatal interval prior to reconnection. Furthermore, the microbial profiles of the S1R56 and S7R56 reconnected segments were highly similar to each other on day 56 (Supplementary Figure 2F and Table 3).

### Delayed Exposure to Digesta Influences Colonization by Beneficial Bacteria

**FIGURE 4:**
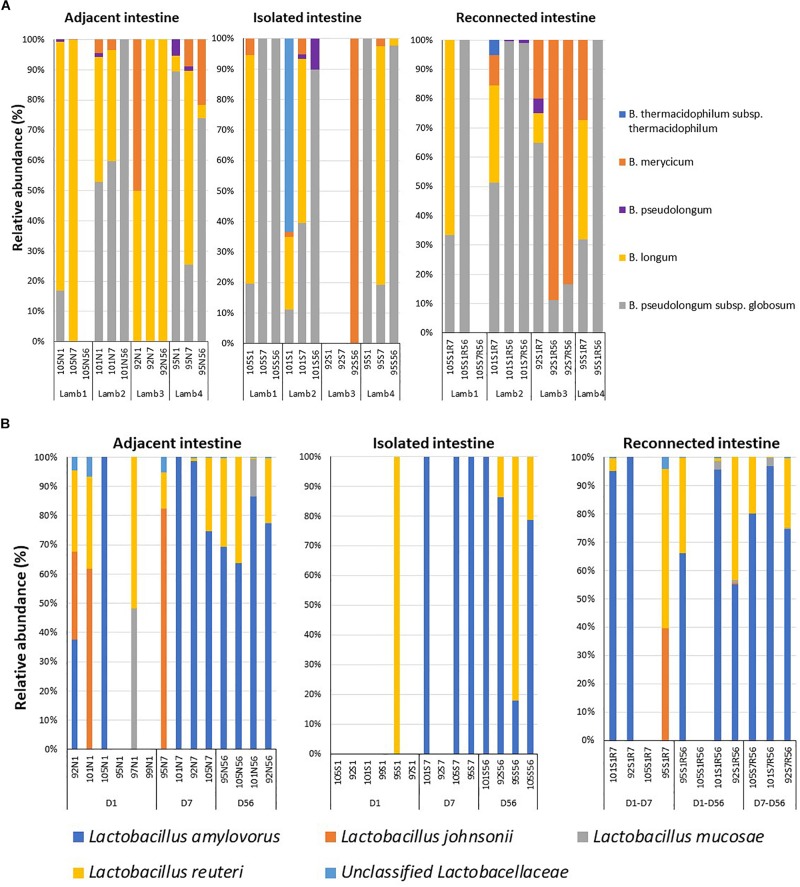
Dysbiosis of the beneficial bacterial community in subsections of the surgically isolated intestinal segments. **(A)** 16S amplicon sequencing based profiling of the genus *Bifidobacterium* composition. **(B)** Species-level composition of the genus *Lactobacillus*. N1 –adjacent intact intestine sampled on day 1; S1 – surgically isolated intestine sampled on day 1; N7 –adjacent intact intestine sampled on day 7; S7 – surgically isolated intestine sampled on day 7; S1R7 – surgically isolated intestine subsection reconnected on day 1 and sampled on day 7; N56 –adjacent intact intestine sampled on day 56; S56 – surgically isolated intestine sampled on day 56; S1R56 – surgically isolated intestine subsection reconnected on day 1 and sampled on day 56; S7R56 – surgically isolated intestine subsection reconnected on day 7 and sampled on day 56.

Early microbial dysbiosis is often linked to an altered abundance of beneficial bacterial populations, including *Bifidobacterium* and *Lactobacillus*. Universal bacterial primers used in the present study did not efficiently amplify the genus *Bifidobacterium*, which belongs to the phylum *Actinobacteria* (Supplementary Figure 3). Therefore, *Bifidobacterium* genus-specific primers were used to profile the composition of this beneficial bacterial population. A total of 13 bifidobacterial OTUs could be assigned to five different species when lamb intestinal samples were analyzed (Figure 4A). *B. pseudolongum* subsp. *globosum* (day 1 – 39.7 ± 19.9%, day 7 – 21.3 ± 14.2%, day 56 – 43.5 ± 25.7%) and *B*. *longum* (day 1 – 44.8 ± 15.9%, day 7 – 75.2 ± 15.3%, day 56 – 26.1 ± 24.6%) were the most frequent and most abundant *Bifidobacterium* species identified in samples collected from the adjacent intestine. *B. pseudolongum* subsp. *globosum* (day 1 – 32.7 ± 22.7%, day 7 – 39.7 ± 21.6%, day 56 – 71.8 ± 24.1%) was also the most abundant *Bifidobacterium* identified in samples from the isolated intestinal segments. The relative abundance of *B*. *longum* in the isolated intestinal segment was numerically high on day 1 (24.7 ± 17.7%) and day 7 (33.1 ± 19.7%) but could be identified in only one animal on day 56 (0.6%). *B*. *thermacidophilum* subsp. *thermacidophilum* (15.9%) was observed in the isolated intestinal segment of only one animal on day 1. Although reconnection of the isolated intestinal segments facilitated colonization by *B. pseudolongum* subsp. *globosum* (S1R7 – 45.4 ± 7.9%, S1R56 – 77.7 ± 22.2%, S7R56 – 38.5 ± 30.5%), *B*. *longum* was only observed in S1R7 (37.7 ± 11.7%) but not in S1R56 and S7R56 reconnected intestinal segments (Figure 4A).

When the active *Bifidobacterium* densities were estimated, adjacent intestine had higher *Bifidobacterium* densities throughout the experimental period than either the surgically isolated intestinal segments or the reconnected intestinal segments (Table 2). On day 56, *Bifidobacterium* density in the reconnected intestinal segment was significantly (*P* < 0.01) higher than the age-matched isolated intestinal segment but remained significantly (*P* < 0.01) lower than adjacent intestine (Table 2).

Species level composition of the genus *Lactobacillus* was also analyzed by extracting sequence information from the total bacterial profiles generated using universal bacterial primers. *L. amylovorus* was the most abundant and frequent species identified in samples from all intestinal segments (Figure 4B) but *L. amylovorus* was not detected in any samples collected from the day 1 isolated intestinal segments. *L*. *johnsonii* and *L*. *mucosae* were only detected in samples from adjacent intestine and reconnected intestinal segments (Figure 4B). Associated with these compositional differences, the density of *Lactobacillus* also varied when comparing adjacent intestine and isolated intestinal segments (Table 2). *Lactobacillus* density in the adjacent intestine was significantly (*P* < 0.01) higher than that in the isolated intestinal segments throughout the entire experimental period. Relative to the adjacent intestine, *Lactobacillus* density was significantly (*P* < 0.01) lower in the reconnected intestinal segments on day 7 but not on day 56 (Table 2).

## Discussion

A new animal model was developed to study perturbations in the regional microbial community in the distal small intestinal following a short- (24 h) and long-term (7 days) delay in exposure to ingesta following birth. A surprising observation in the present study was establishment of an active and diverse bacterial community within 24 h after birth within the intestinal segments surgically isolated *in utero* (Figure 2B). This bacterial community was similar to the pioneer community colonizing the adjacent intact intestine and remained stable in the isolated segment throughout the 8 weeks study period. Recent studies reported the presence of bacteria in the fetal environment (Aagaard et al., 2014; Collado et al., 2016; Zheng et al., 2017), which created debate regarding the initial gut colonization process (Perez-Muñoz et al., 2017; Walker et al., 2017). We confirmed the ovine fetal small intestine and fetal environment were sterile at the time of surgery during the third trimester of pregnancy (Malmuthuge and Griebel, 2018). Supporting our observations, Leiby et al. (2018) recently reported the absence of detectable bacterial DNA in human placenta. Analysis of small intestinal samples, collected from calves within 5 min of birth revealed colonization by an active, dense and complex bacterial community (Malmuthuge, 2016). These findings suggested colonization of the mammalian GIT begins during the birthing process, with the fetus exposed *in utero* to maternal and environmental microbiota following cervical dilation and rupture of amniotic membranes. Similar to neonatal calves (Malmuthuge, 2016; Malmuthuge et al., 2019), the microbiome in the small intestine of newborn lambs was dominated by *Proteobacteria* with a transition to *Firmicutes* with increasing age and exposure to ingesta. *Massilia* (phylum *Proteobacteria*) was one of the abundant bacterial genera observed in the small intestine of newborn lambs, including both the adjacent intact small intestine and the surgically isolated intestinal segments (Supplementary Figure 1A).

Intestinal segments surgically isolated *in utero* had no physical connection with the lumen of adjacent intestine in the newborn lamb, preventing access to ingesta during and after birth (Figure 1). These intestinal segments do, however, retain blood flow and lymphatic drainage through the mesenteric attachment. We hypothesized the surgically isolated intestinal segments would provide a gnotobiotic region that would not be colonized by maternal and environmental bacteria during birth and could therefore be used to study the effects of delayed exposure to ingesta and microbiota during the post-partum period. An active bacterial community was, however, observed in the isolated intestinal segments within 24 h post-partum and this community included both *Bifidobacterium* and *Lactobacillus* (Table 2). Lymph drains away from the intestine, while blood flows both to and from the intestine. One possibility is that bacteria may reach the surgically isolated intestinal segments through blood. The intestinal mucosal epithelial barrier is highly permeable during the first 6 h after birth (Bush and Staley, 1980) and systemic bacterial infections are common in colostrum-deprived ruminants (Stilwell and Carvalho, 2011). Furthermore, it has been suggested that *Mycobacterium avium* subsp. *paratuberculosis*, a bacterial pathogen of sheep and cattle, can translocate from intestinal tissue into the intestinal lumen when intestinal barrier function is compromised (Koets et al., 2015). The aerobic bacterial group, *Massilia*, has previously been isolated from the blood of humans (Lindquist et al., 2003), indicating they can cause a bacteremia. It remains to be determined, however, whether the diverse pioneer microbial community observed at 24 h post-partum in surgically isolated intestinal segments, including *Bifidobacterium* and *Lactobacillus*, was present in the blood of lambs at the time of birth.

While bacterial colonization was observed in the surgically isolated intestinal segments, this community had a 90% reduction in bacterial density and the composition was altered when compared to age-matched adjacent intestine. Both reduced density and altered composition are defining characteristics of microbial dysbiosis. Moreover, a bacterial community similar to the pioneer bacteria of newborn lambs (*Proteobacteria*-dominant) was maintained at a relatively constant density in the isolated intestinal segment over a 2-month period (Figure 2B). Two notable features of neonatal microbiomes are high individual animal variation and rapid temporal changes in composition within outbred species, such as humans and large animals (Yatsunenko et al., 2012; Dominguez-Bello et al., 2016; Malmuthuge et al., 2019). Thus, studies on the neonatal gut microbiota of outbred mammals always requires a large number of replicates to identify statistical differences among treatments (Dominguez-Bello et al., 2016). Moreover, dynamic changes in the indigenous microbial community may interfere with the effects of exogenous microbiota, especially when studying interventions to manipulate the early gut microbial colonization process. The surgically isolated intestinal segments in the present study provided, however, a stable microbial environment (Figure 3A). This stable environment can now be used to investigate the effect of both prolonged microbial dysbiosis and subsequent interventions to alter the microbial community on development of mucosal immune function.

Early microbial dysbiosis is often associated with variations in the diversity and/or density of beneficial bacterial groups, such as *Bifidobacterium* and *Lactobacillus*. Cesarean delivery (Dominguez-Bello et al., 2010; Sirilun et al., 2015), bottle/formula feeding (Tannock et al., 2013), and antibiotic use (Korpela and de Vos, 2016) in humans as well as colostrum deprivation (Malmuthuge et al., 2015; Song et al., 2019) and antibiotic use (Oultram et al., 2015) in neonatal calves have been associated with significant alterations in beneficial bacterial communities. The surgically isolated intestinal segments in the present study also displayed significant variation in the density and diversity of the beneficial bacteria, *Bifidobacterium* and *Lactobacillus*, when compared to normal intestine (Figure 4). It remains to be determined whether these microbial perturbations are associated with significant alterations in mucosal gene expression but this model provides an opportunity to determine if specific *Bifidobacterium* or *Lactobacillus* species significantly alter mucosal function in neonatal mammals. Early microbial dysbiosis can have a long-term impact on the host and subsequent bacterial colonization (Subramanian et al., 2015). Therefore, early interventions to restore beneficial microbiota of neonates, prior to establishment of a stable adult microbiome, has been proposed as a way to prevent undesirable health outcomes linked to altered neonatal gut microbiota (Milani et al., 2017). Introduction of maternal birth canal microbiota immediately after birth was partially effective in restoring beneficial bacteria in cesarean-delivered human infants (Dominguez-Bello et al., 2016), implying early exposure during initial colonization may be an effective early intervention. This study investigated fecal microbiota of infants but not the impact of early interventions on regional enteric microbiota. Neonatal enteric microbiota is distinct from maternal rectal and birth canal bacteria (Malmuthuge, 2016) and needs to be studied using regional intestinal samples. Access to regional intestinal samples is limited but our model provides an appropriate tool to repeatedly sample and analyze the regional microbiota over the more prolonged neonatal developmental period of large mammals.

In the present study, isolated intestinal segments with a stable and dysbiosed microbial community were exposed to autogenous ingesta containing the complete regional gut microbiome (autochthonous microbiota). This was achieved by reconnecting subsections of the isolated intestinal segments to the adjacent small intestine and this reconnection successfully restored total bacterial density and composition at the phylum level (Figure 2C). Bacteriotherapy (fecal matter transplantation - FMT) is one intervention currently used to transfer complete gut microbiome from healthy individuals to individuals with dysbiosed microbiomes (Choi and Cho, 2016). FMT is being used primarily in adults with a stable (highly resilient) microbiome, thus, results are not consistent. Use of super-donors to create full restoration of dysbiosed microbiome was recently suggested as an alternative due to inconsistent and short-term restoration observed with FMT (Wilson et al., 2019). Although individual lambs in the present study displayed distinct recovery patterns during the 7–8 weeks recovery period, bacterial communities established in the reconnected intestinal segments displayed a high level of similarity when compared to the adjacent intact intestine on day 56. These results support the conclusion that exposure to autochthonous gut microbiome during early life can, to a large extent, effectively restore a dysbiosed microbiome.

In the present study, we delayed exposure to ingesta by either 1 or 7 days post-partum, which created relatively short-term dysbiosis. However, beneficial bacterial communities (*Bifidobacterium* and *Lactobacillus*) were not fully restored even after this relatively short-term microbial dysbiosis (Figure 4 and Table 2). These two beneficial bacterial genera are often highly abundant in neonates and gradually decline with decreasing milk consumption and increasing consumption of solid food (Uyeno et al., 2010; Yatsunenko et al., 2012; Oikonomou et al., 2013). Thus, it is important to understand if complete restoration of beneficial bacteria through early interventions will require fortification (prebiotics, probiotics, synbiotics) together with exposure to the appropriate regional microbiota.

## Conclusion

The present study created microbial dysbiosis within intestinal segments surgically isolated *in utero* and this dysbiosis was characterized by a significant and stable reduction in both bacterial diversity and density. The stable intestinal environment was colonized by pioneer bacteria (*Proteobacteria*), which then persisted for 2 months. This model system provides an opportunity to study the effects of early microbial dysbiosis throughout the prolonged neonatal period of large mammals. Further, this model provides the opportunity for repetitive sampling to study the kinetics of both microbial and immune responses to therapeutic microbial interventions. Restoration of the dysbiosed microbial community was achieved to a large extent by reconnecting subsections of the surgically isolated intestinal segment to adjacent intact intestine. However, 2 months after exposure to autochthonous microbiome the beneficial bacterial groups remained significantly altered in the reconnected subsections of intestinal segments relative to adjacent intact intestine. Future studies are now possible with this model to identify more effective means to restore beneficial bacterial populations that are highly abundant in the neonatal intestine. Dividing *in utero* isolated intestinal segments into multiple, independent subsections facilitates a comparison of multiple therapeutic strategies within a genetically identical GIT environment. This genetically identical environment should reduce the inherent microbiome variability observed among individuals in an outbred species and reduce the number of animals required for each study.

## Data Availability

The datasets generated for this study can be found in NCBI SRA, https://www.ncbi.nlm.nih.gov/sra/PRJNA507407.

## Ethics Statement

All experimental protocols were reviewed and approved by the University of Saskatchewan Animal Care Committee (AUP20160105), which follows the guidelines of the Canadian Council on Animal Care.

## Author Contributions

NM designed the animal experiments, performed the nucleic acid extraction, PCRs, and qPCRs, analyzed and interpreted 16S amplicon sequencing data, and wrote the manuscript. PG involved in designing the animal experiments, data interpretation, and manuscript editing. Both authors read and approved the final manuscript.

## Conflict of Interest Statement

The authors declare that the research was conducted in the absence of any commercial or financial relationships that could be construed as a potential conflict of interest.

## Footnotes

1 https://www.ncbi.nlm.nih.gov/sra/PRJNA507407
