# Supplementary material for: A Novel Animal Model for Regional Microbial Dysbiosis of the Pioneer Microbial Community
Source: Front Microbiol. 2019 Jul 24;10:1706. doi: 10.3389/fmicb.2019.01706 (PMC6668574; doi:10.3389/fmicb.2019.01706)
Supplement: Supplementary file 1 [file Data_Sheet_1.PDF]

Supplementary Figure Legends

Supplementary Figure 1. Bacterial diversity of adjacent, isolated and reconnected intestinal segments on days 1, 7, and 56 post-partum. **(A)** Intestinal bacterial composition at the genus level. **(B)** Mean alpha diversity indices of intestinal bacterial communities. N1 – adjacent intestine sampled on day 1; S1 – isolated intestine sampled on day 1; N7 – adjacent intestine sampled on day 7; S7 – isolated intestine sampled on day 7; S1R7 – intestinal segment reconnected on day 1 and sampled on day 7; N56 – adjacent intestine sampled on day 56; S56 – isolated intestine sampled on day 56; S1R56 – intestinal segment reconnected on day 1 and sampled on day 56; S7R56 – intestinal segment reconnected on day 7 and sampled on day 56.

Supplementary Figure 2. OTU-based clustering of microbial profiles generated from adjacent intestine and intestinal segments. **(A)** Principle coordinate analysis of all microbial communities (all time points and all intestinal regions). **(B)** Comparison of three intestinal regions within each time point. **(C)** Comparison of microbial profiles generated from adjacent and reconnected intestinal segments within each time point. **(D)** Comparison of microbial profiles generated from adjacent and isolated intestinal segments. **(E)** Comparison of microbial profiles generated from adjacent intestine and isolated and reconnected intestinal segments. **(F)** Comparison of microbial profiles between intestinal segments reconnected on day 1 and day 7. **(G)** Clustering of microbial profiles within each animal using ρ^2 generated through pairwise comparisons of individual microbial profiles.

Supplementary Figure 3. Relative abundance of phylum *Actinobacteria* in the microbial profiles generated from normal intestine and intestinal segments.

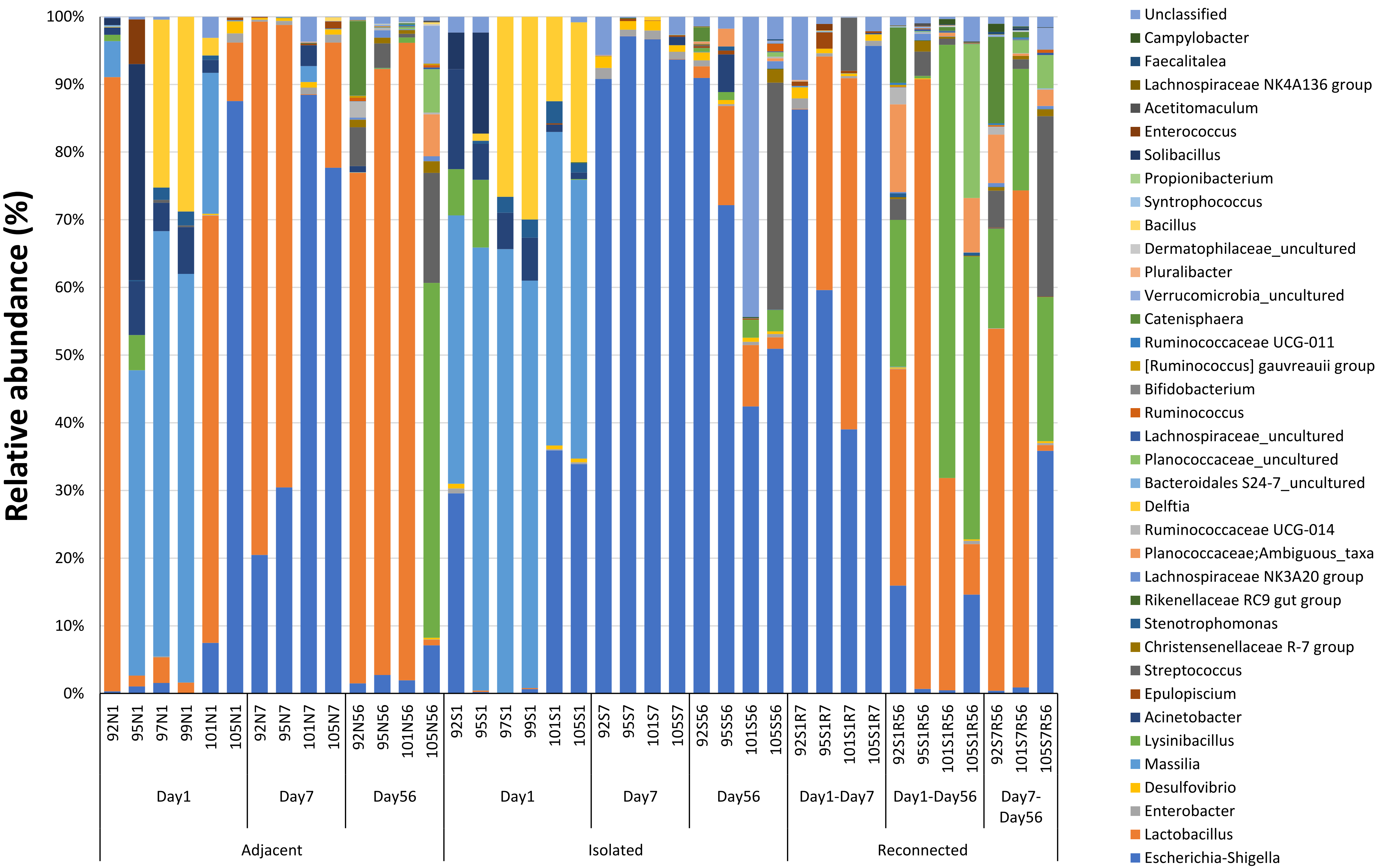

Supplementary Figure 1A

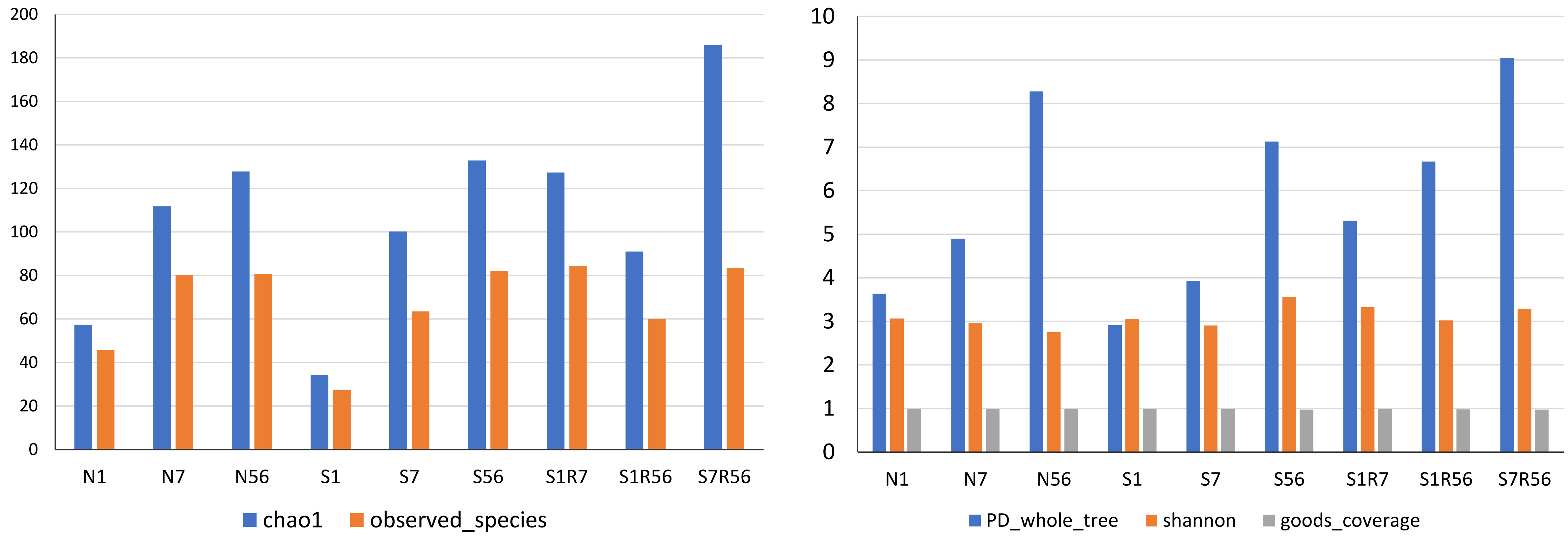

Supplementary Figure 1B

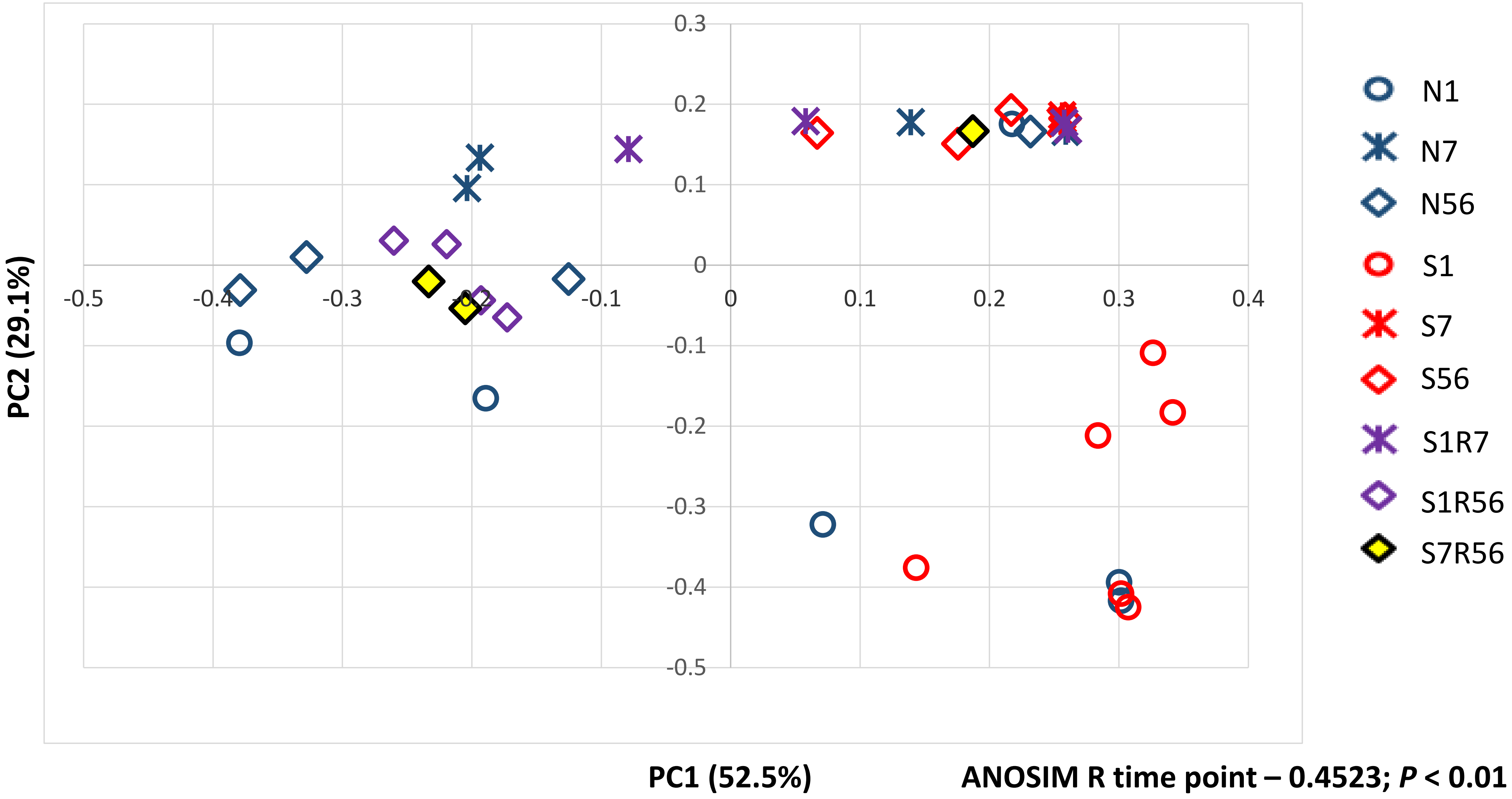

ANOSIM R time point – 0.4523; *P* < 0.01
ANOSIM R intestinal region – 0.1977; *P* < 0.01

Supplementary Figure 2A

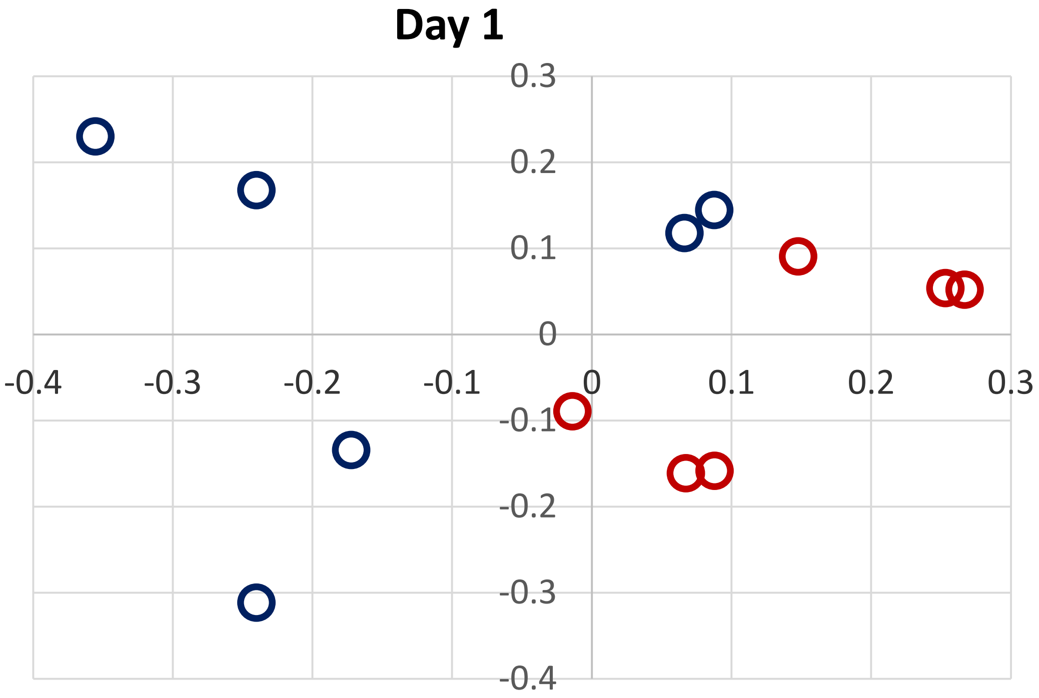

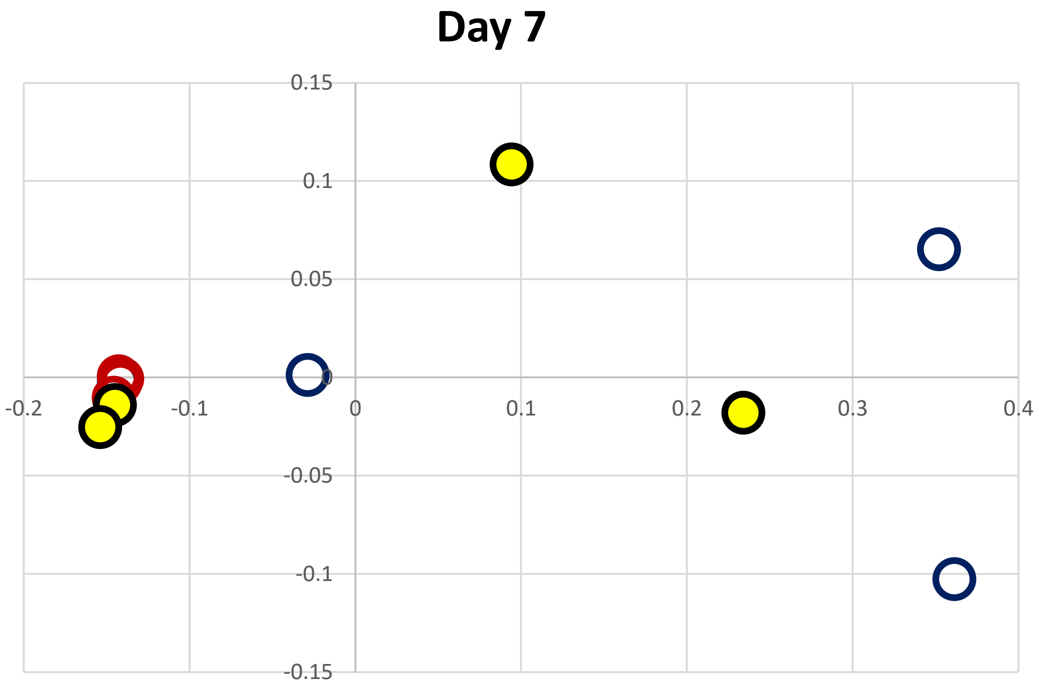

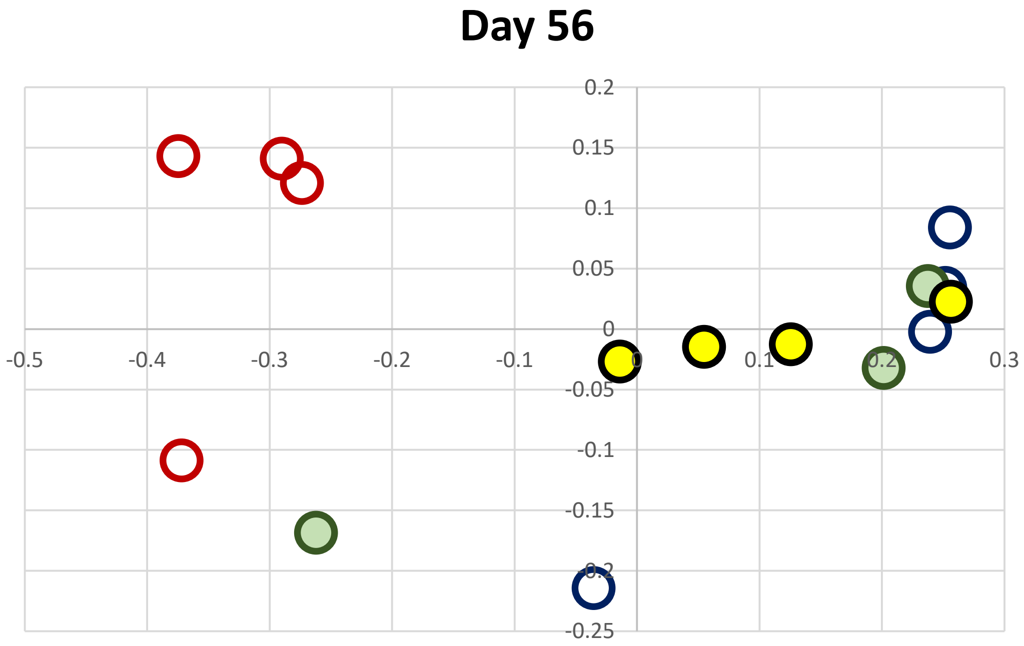

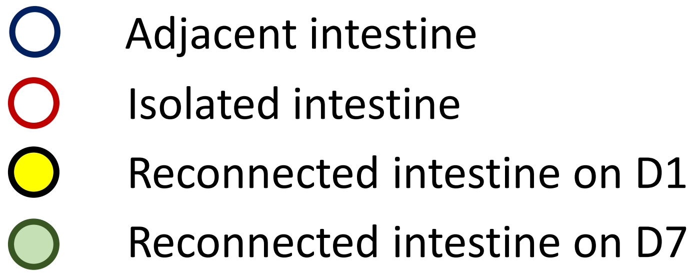

Supplementary Figure 2B

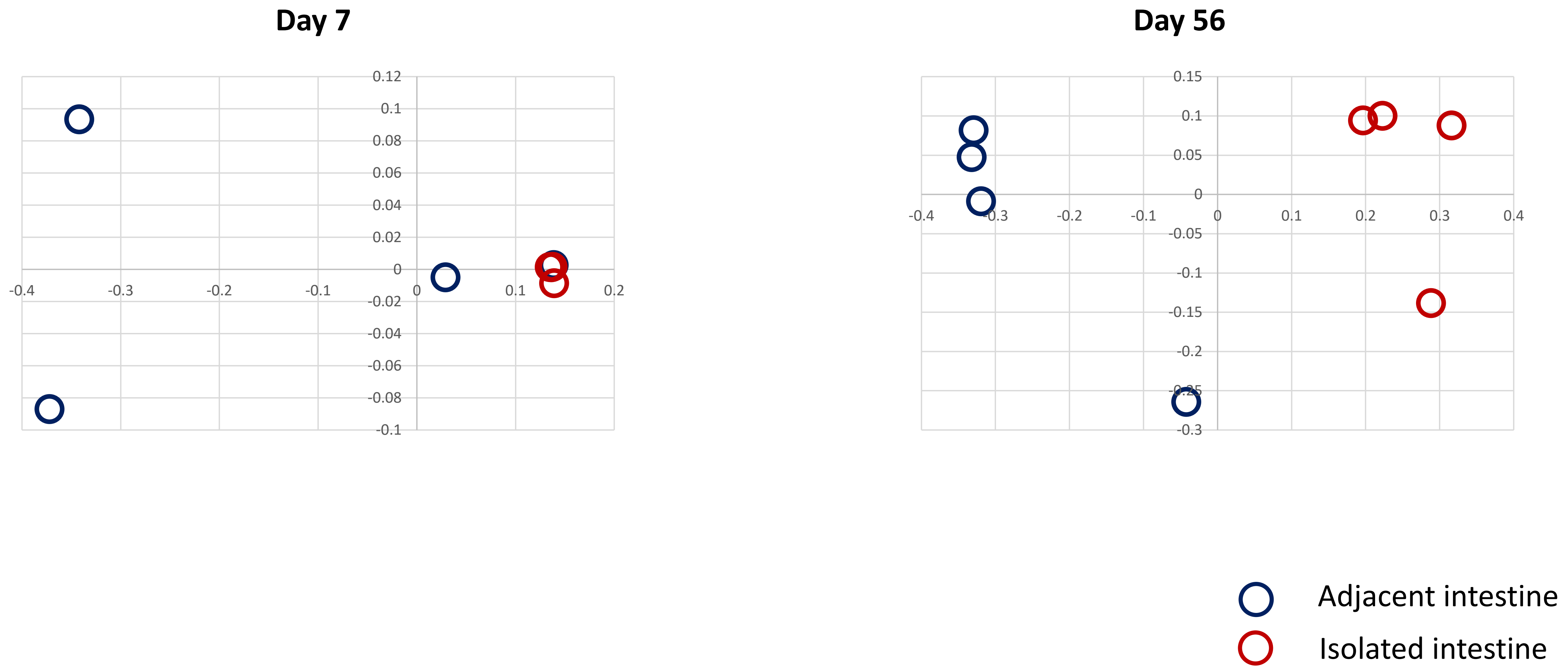

Supplementary Figure 2C

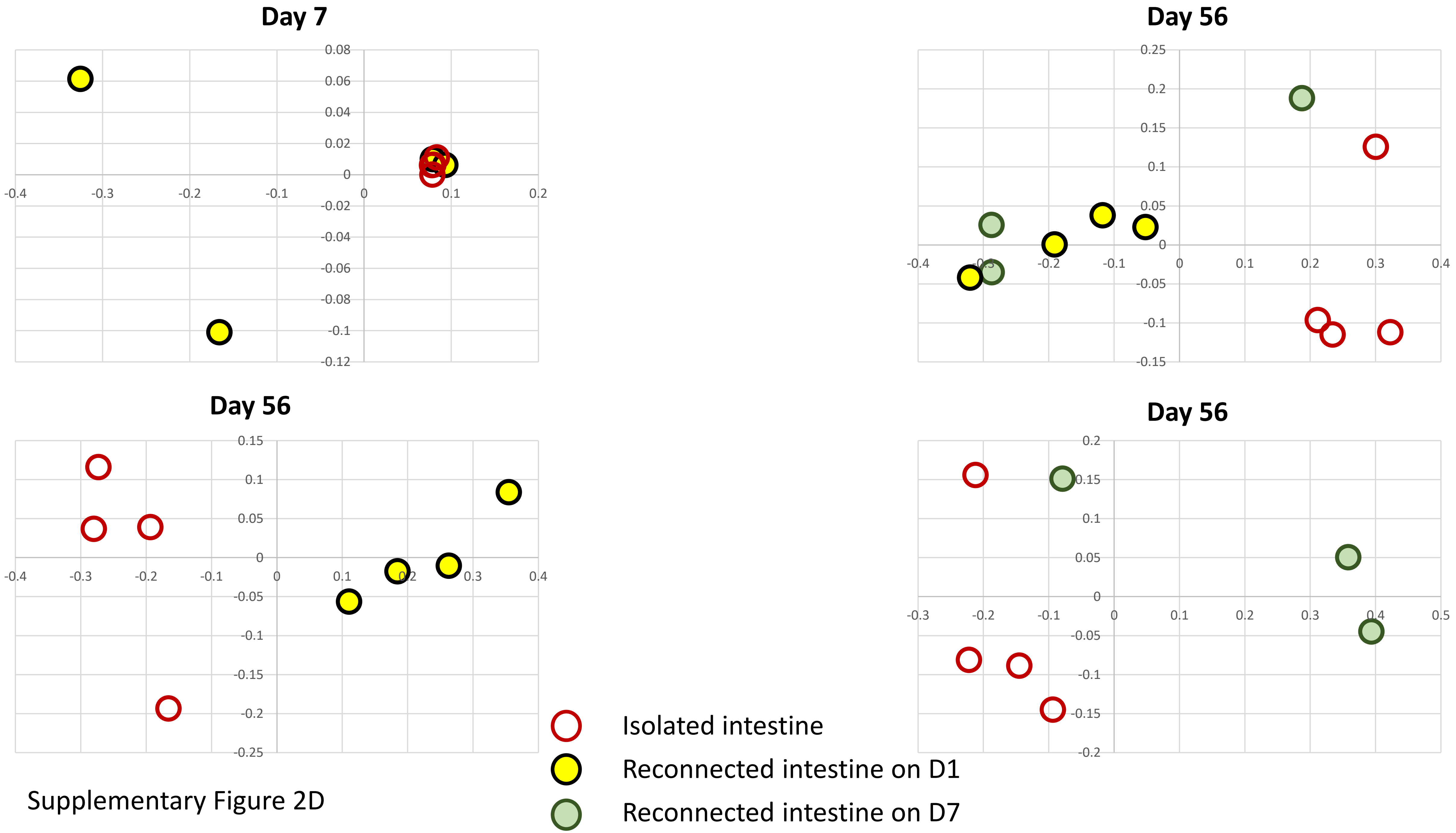

Supplementary Figure 2D

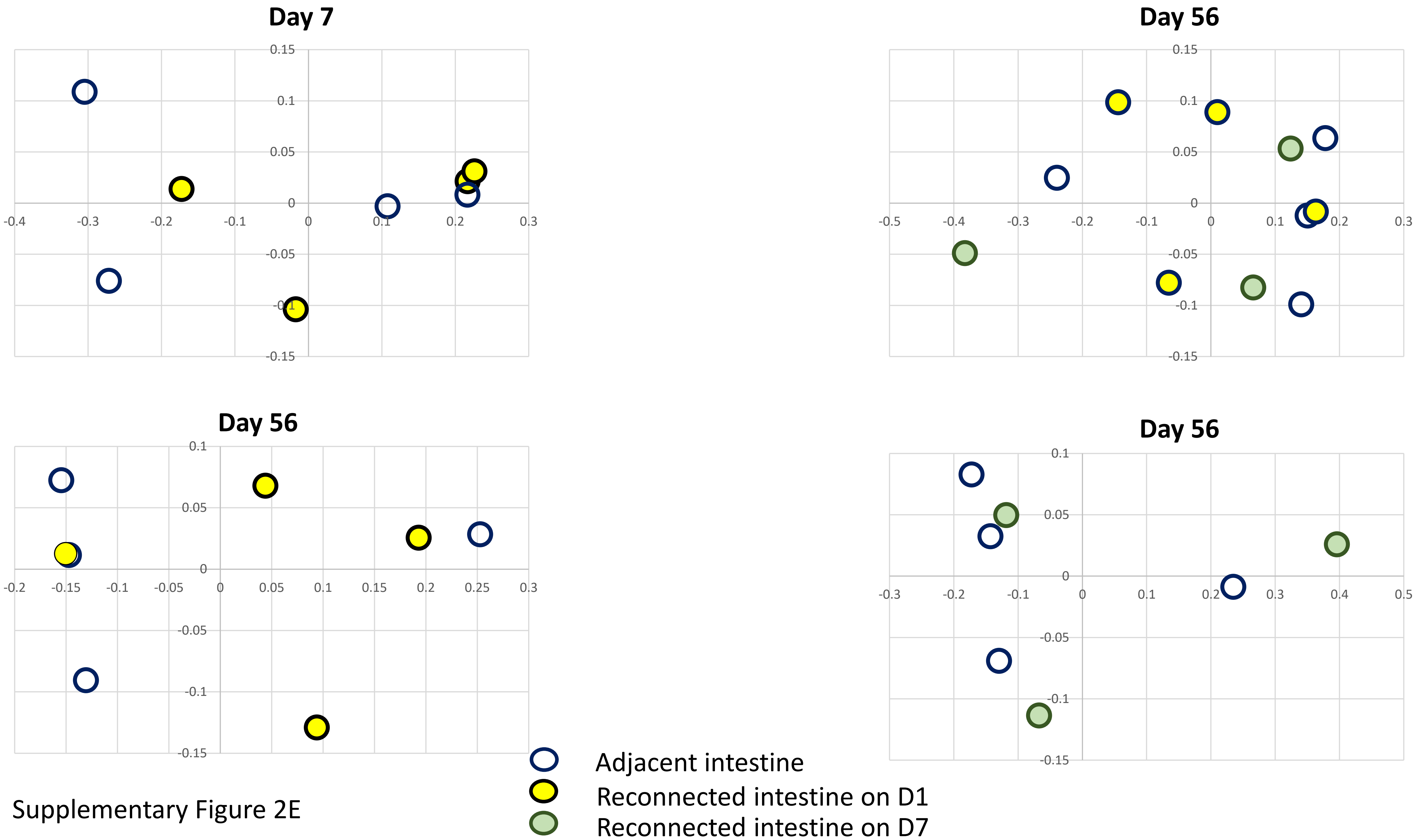

Supplementary Figure 2E

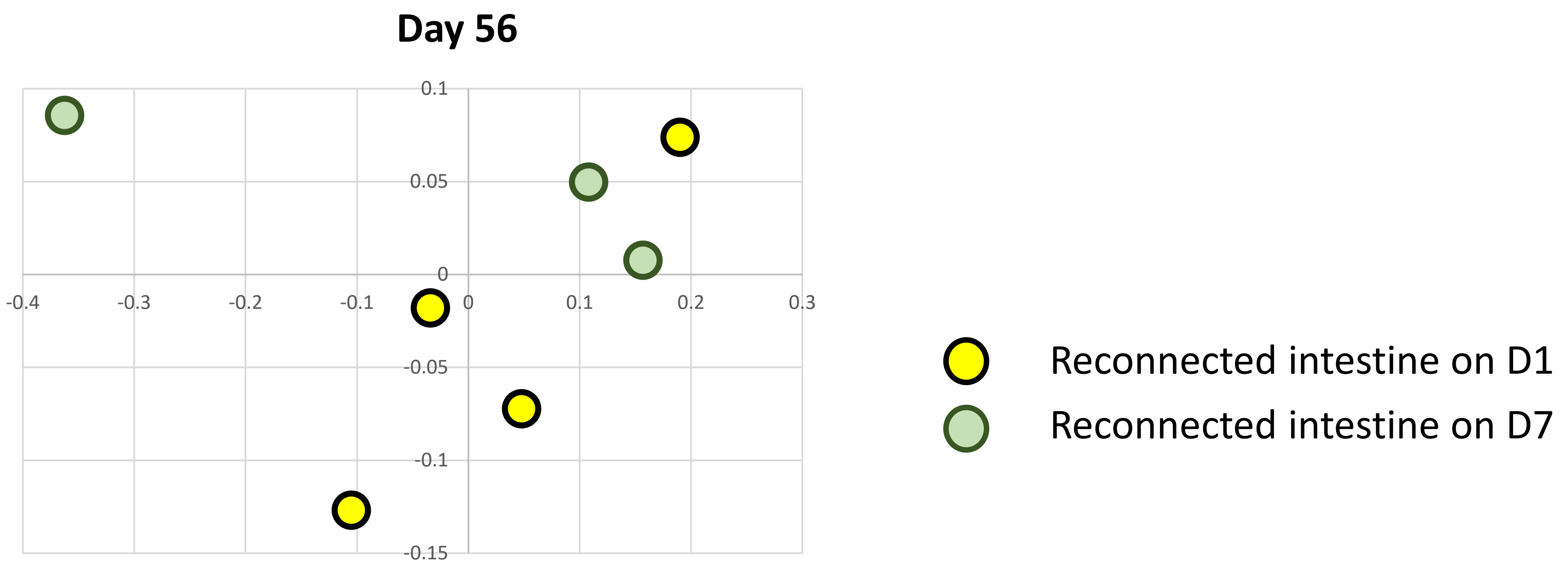

Supplementary Figure 2F

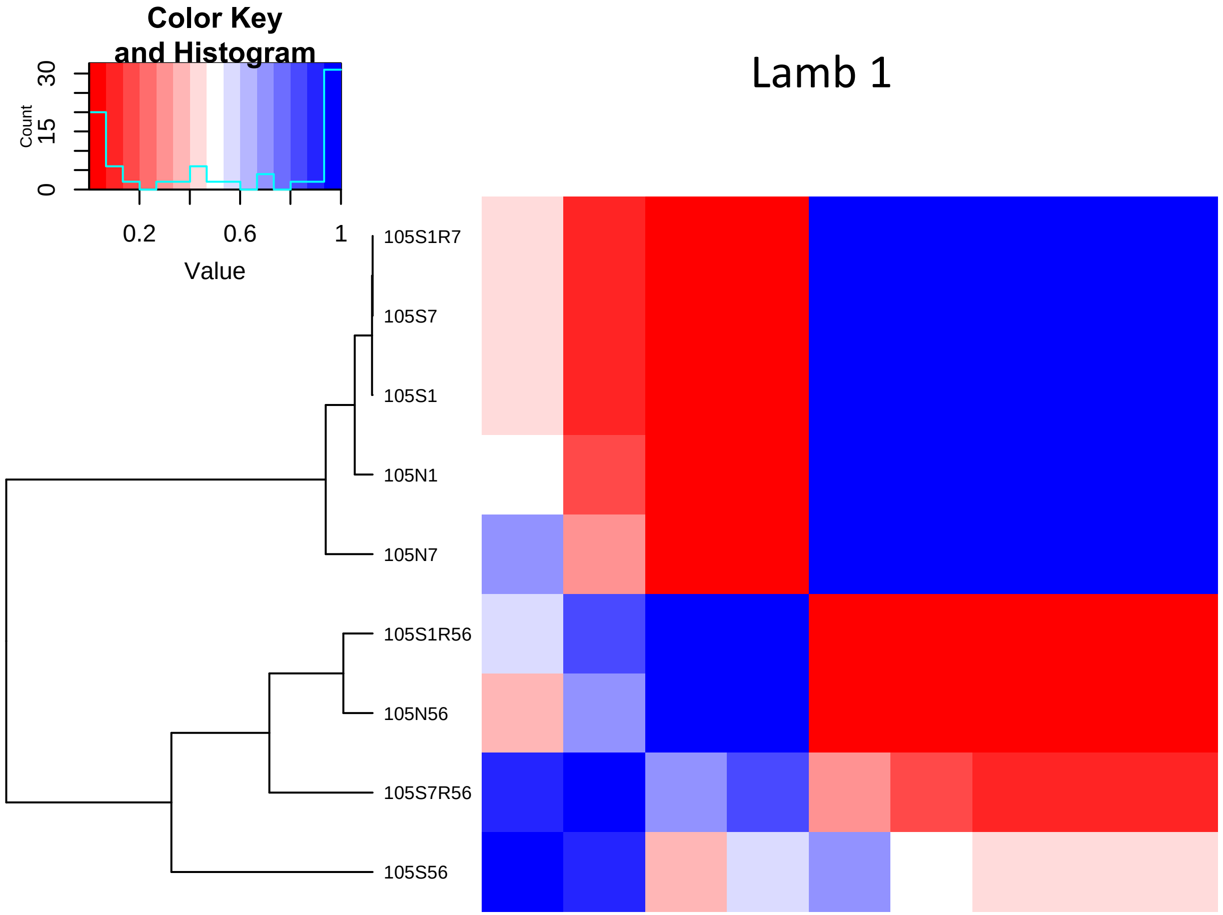

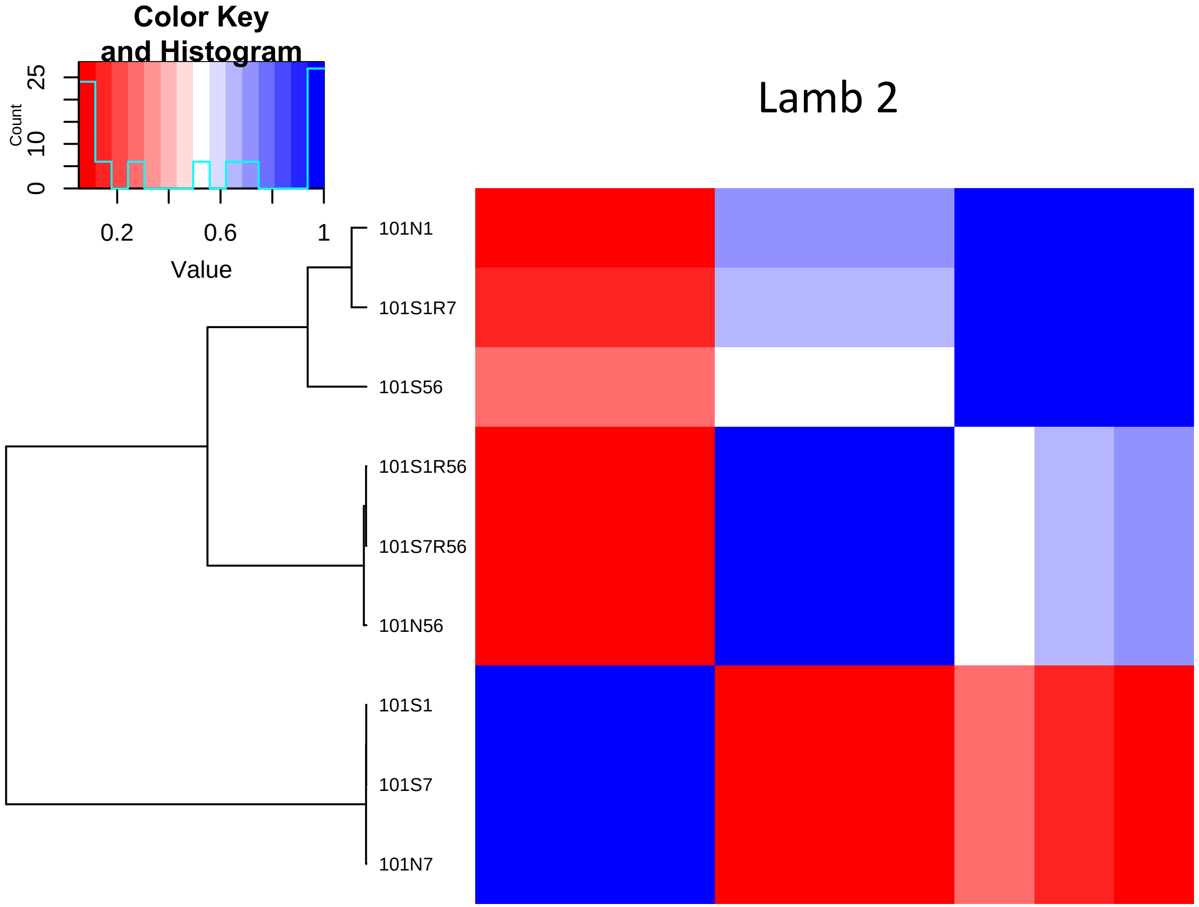

Supplementary Figure 2G

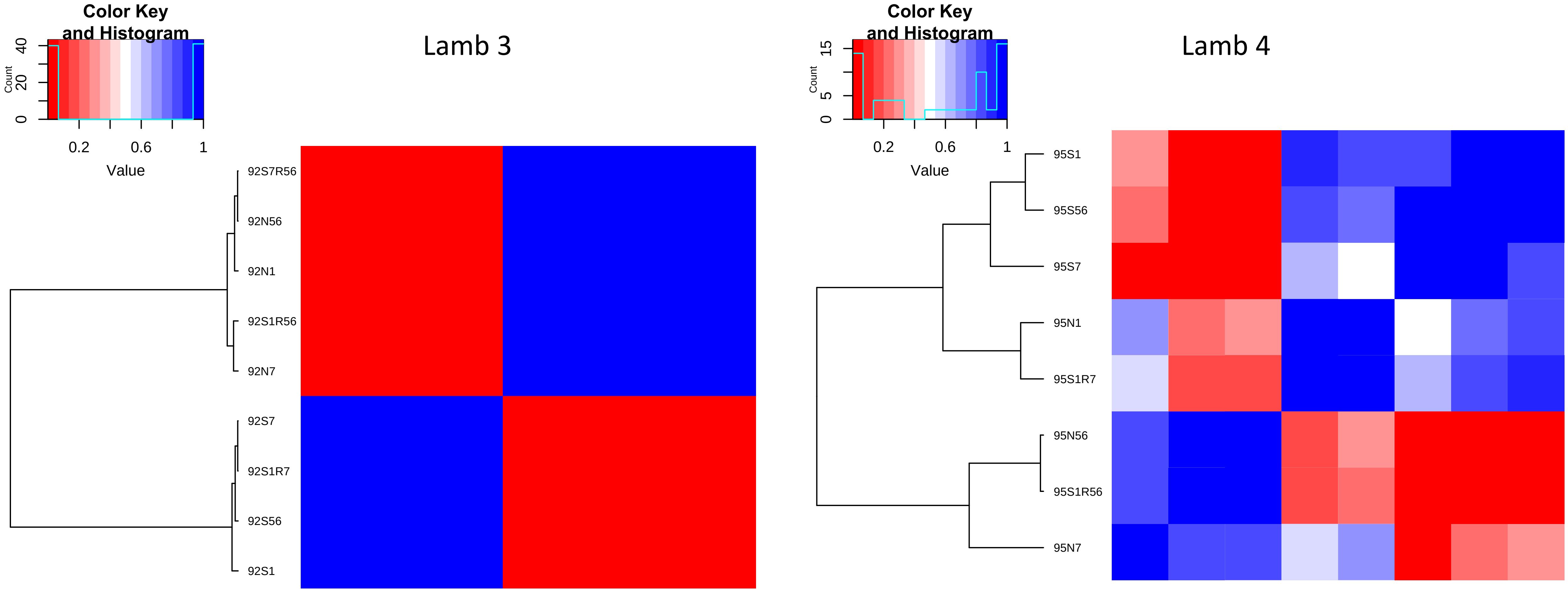

Supplementary Figure 2G

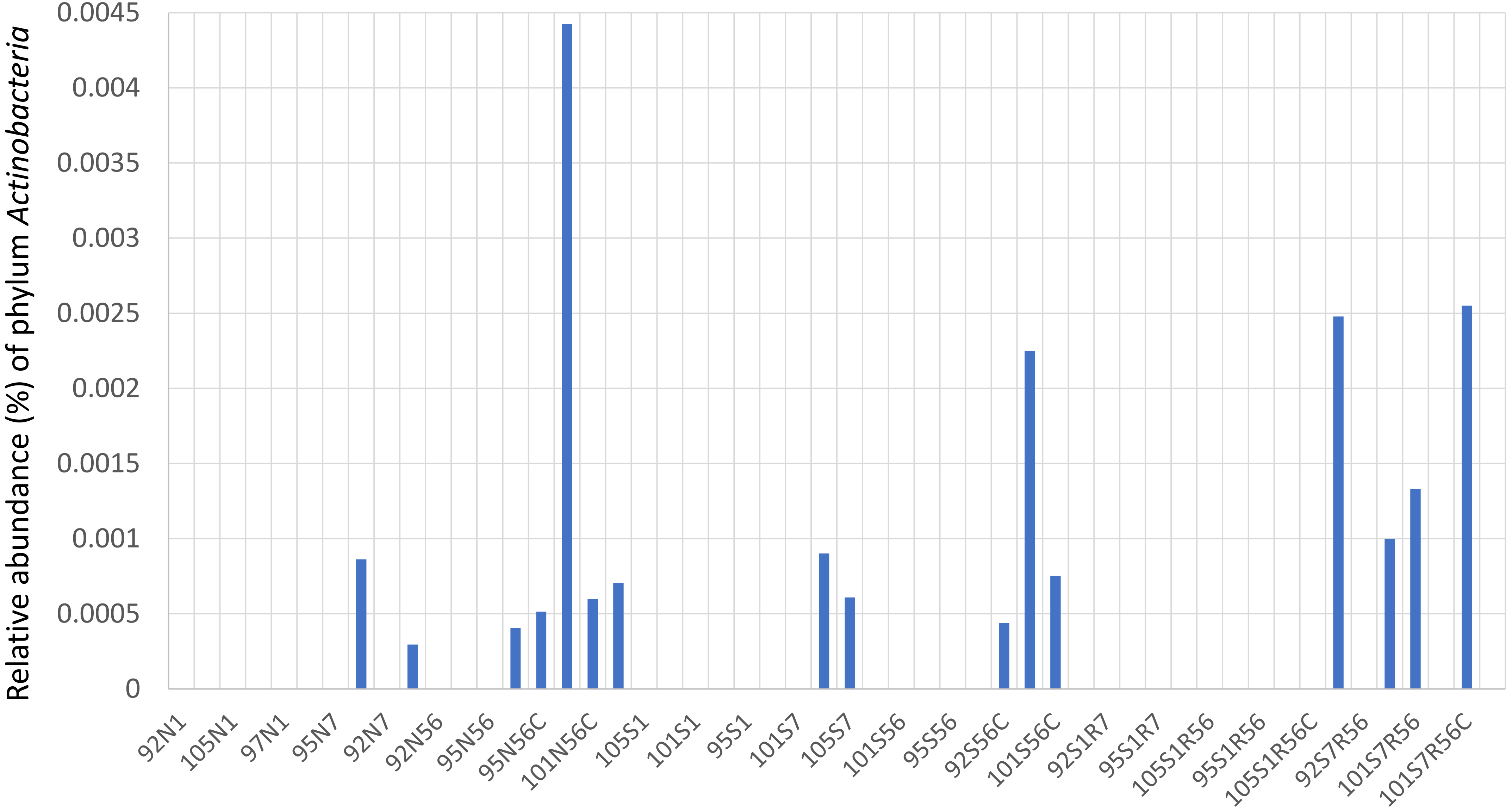

Supplementary Figure 3
